# How to deal with future uncertainty? An empirical study of undergraduates based on the theory of motivation information management

**DOI:** 10.3389/fpsyg.2025.1537814

**Published:** 2025-05-29

**Authors:** Zhuowen Feng, Chuyu Guo, Guancheng Wang, Chunyan Mo

**Affiliations:** ^1^College of Literature and News Communication, Guangdong Ocean University, Zhanjiang, Guangdong, China; ^2^College of Electronic and Information Engineering, Guangdong Ocean University, Zhanjiang, Guangdong, China; ^3^Communist Youth League, Guangdong Ocean University, Zhanjiang, Guangdong, China

**Keywords:** Theory of Motivated Information Management, uncertainty, undergraduates, graduation anxiety, information seeking

## Abstract

Based on the Theory of Motivated Information Management (TMIM), this study examines how undergraduates manage information related to post-graduation pathways, with a particular focus on their interactions with teachers, family, and friends. In addition, it explores how individuals perceive and process information in uncertain situations, specifically examining undergraduates' behaviors and decision-making regarding employment and further education. Furthermore, this study sheds light on the decision-making processes of students facing graduation choices, particularly in terms of information management. It addresses a gap in the application of the TMIM model within the context of college graduation anxiety research, assessing its suitability as a decision-making model for information seeking related to graduation anxiety. The findings demonstrate the effectiveness of the TMIM framework in predicting information-seeking strategies, thereby supporting various application scenarios of information-seeking models for undergraduates.

## 1 Introduction

The growing economies of developing countries enhance competitiveness across various industries while also generating new business sectors and opportunities. In recent years, China has witnessed a significant surge in the number of college graduates, resulting in increasingly competitive and challenging employment prospects for students. The number of college graduates in 2023 has been reported to have increased to 11.58 million, marking an increase of 820,000 compared to 2022 (Liu, 2023). In contrast, the employment rate of university graduates in China in 2024 was only 55.5%, a decrease of 2.1 percentage points compared to 2023. Furthermore, college students who graduated in the 1990s can be said to be almost 100% employed, so the issue of “employment difficulties” has become a hot issue in society today.

In addition, forecasts indicate that this number will increase further to 11.87 million by 2024. Evidently, undergraduates are confronted with cut-throat competition and a demanding job market, leading to more intricate and diverse psychological issues related to employment. The overall pressure to find employment is considerable (Song and Ma, 2021), with stress levels exceeding the medium range (Meng et al., 2022). Meanwhile, as employment competition intensifies, the trend of “slow employment” among undergraduates has gained prominence, and many graduates opt to further their education or seek stable positions through exams. For example, becoming a civil servant is one of the popular choices for undergraduates due to its stability. Unlike the civil service, in recent years, many companies have seen a decline in profitability, resulting in unstable employee income and the risk of layoffs, which has indirectly contributed to the rising phenomenon of taking the civil service exam (Cuojia and Guisang, 2020). In 2023, the number of civil service applicants in China has reached 2.5 million, marking a 22% increase from the previous year and continuing the upward trend over five years. Similarly, the number of postgraduate applicants is also steadily increasing. In 2022, China had 10.76 million college graduates, of whom 4.57 million applied for postgraduate and graduate examinations (Tian and Gong, 2023). Concurrently, postgraduate education remains a dominant pathway for deferring employment pressures. This convergence of hypercompetitive career pathways and deferred employment pressures has inadvertently intensified graduates' psychological burdens, transforming career planning into a pervasive anxiety. The heightened pressure of securing employment can induce fear and uncertainty among graduates regarding their future. Negative emotions include anxiety, a sense of loss, excessive stress, and self-doubt, potentially affecting the physical and mental well-being of undergraduates. Consequently, addressing the psychological aspects of employment for undergraduates has become an urgent and critical issue.

Recently, numerous researchers have focused on this topic. Zhao (2023) comprehensively evaluated the employment psychological status of modern undergraduates using the AHP-TOPSIS evaluation method. The findings reveal that, in general, the psychological state of employment for undergraduates is significantly affected by the support provided by schools and families. Factors such as economic and emotional support from families, the cultivation of the school's employment concept, employment-focused lectures, and the development of related courses can effectively alleviate undergraduates' employment anxiety and foster their mental health development. Additionally, Feng (2023) designed an employment information management system that uses a deep learning framework to address issues within existing employment information management systems. The system collects basic student information through a dedicated module, analyzes and manages student employment situations, and promptly updates universities' employment statuses based on a release module. Based on grounded theory, Tian and Gong (2023) analyzed the motivations of post-graduate students. Their study concluded that initial motivations encompass both rational considerations and emotional aspects, involving both active engagement and passive participation. As the number of postgraduate entrance examinations increases, the evolving integration of internal and external motivations shapes different preferences among candidates. Moreover, Xue (2004) highlighted that both internal and external factors influence motivations and behaviors related to postgraduate entrance exams, with internal factors playing a crucial role. Successful candidates in postgraduate entrance exams are often driven by rational motivations, have clear goals, and are well prepared (Li and Ren, 2013). The research carried out by Li and Ren identified individual characteristics, college teaching environments, academic performance, employment pressure, and parental desires or encouragement as key factors influencing the willingness to take the postgraduate entrance exam.

As graduation approaches, undergraduates are confronted with a range of complex choices and a flood of diverse information. Due to the complexity of the external information environment, employment, entrance information, and the possibility of events or the probability of judgment, as well as personal knowledge of the state of employment, may heighten their uncertainty about the future and influence their communication behavior during various individual information exchanges. This study examines the psychological mechanisms of employment among Chinese university students through a tripartite cultural-institutional lens (Fei et al., 1992): (1) institutional pressures from China's gaokao system, which links elite university access to exam scores, fostering achievement anxiety paradigms; (2) guanxi paradoxes, where career advancement relies on strong-tie networks (family, kinship), creating dual pressures of resource dependency and psychosocial costs; and (3) cultural value constraints, as career decisions balance personal aspirations with familial obligations, a collective logic intensified in high power-distance societies (Hofstede). Cross-cultural analysis reveals that East Asian collectivist contexts require integrating institutional constraints, cultural scripts, and relational networks into analytical (Zhou, 2024) frameworks. The policy recommendations—aligned with the Ministry of Education guidelines' in China—propose a family-school-community support network, contrasting with Western individualized counseling models by prioritizing cultural context in designing the intervention. This indigenous theoretical framework offers novel insights into the dynamics of youth employment in non-Western societies, emphasizing context-sensitive mechanisms of psychological adaptation within high power-distance ecosystems. Therefore, based on the Theory of Motivated Information Management (TMIM), this study aims to analyze various communication behaviors, explore different application scenarios of information-seeking models for undergraduates, and examine the connections between the different stages of the TMIM model in various graduation situations. Additionally, this study starts with the individuals undergraduates could interact with in their daily lives and investigates their information management behavior when facing teachers, family members, and friends. The TMIM has been predominantly applied in family information management and health communication. Previous studies have demonstrated its effectiveness, including investigations into how married couples manage uncertainty about their partner's family health history, parental decision-making in childhood vaccination contexts (Li et al., 2020), information information-seeking processes about retirement preferences (Gettings and Kuang, 2021), and more recently, information management challenges among Chinese single mothers, particularly in navigating cultural pressures and stigma risks (Kuang et al., 2022). This study expands the theoretical boundaries of TMIM by applying it to explore career decision-making processes among Chinese university graduates. This research also has diverse practical implications. First, it contributes to a deeper understanding of the psychological needs of university graduates in building social support networks during future planning, offering critical insights into how young adults navigate uncertainty in transitional life stages. Second, the findings of the research can provide actionable references for key stakeholders—including families, teachers, and friends—to help satisfy graduates' emotional and informational needs in facing future challenges. Finally, the study can also provide empirical foundations for optimizing institutional employment support systems, contributing to the individual development of graduates and the smooth operation of the labor market in China.

## 2 Theory of Motivated Information Management

**Figure 1:**
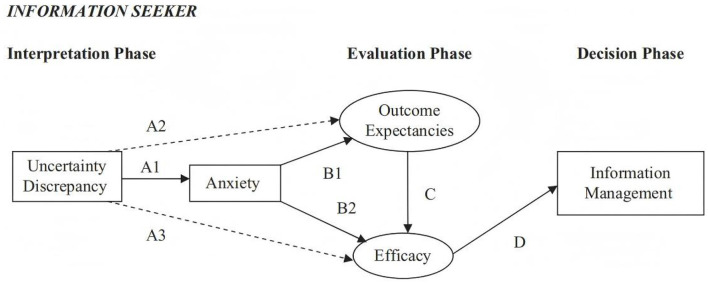
Framework for the original TMIM.

TMIM explains the information management process that people undergo when faced with uncertainty discrepancies. This theory is based on a series of decision-making processes individuals experience when deciding whether to seek, avoid, or share information (Afifi and Weiner, 2004). The original TMIM framework (as shown in Figure 1) divides the decision-making process into three different phases: the interpretation phase, the evaluation phase, and the decision phase, which comprises five variables: uncertainty discrepancy, anxiety, outcome expectancy, efficacy, and information management (Fowler and Afifi, 2011). The interpretation phase involves an individual's awareness of the uncertainty discrepancy; that is, the individual perceives that the level of uncertainty they expect for an important problem does not match their actual level, prompting them to address this discrepancy. Moreover, the discrepancy can also lead to related anxiety and other emotional responses (Afifi and Weiner, 2004). The evaluation phase includes two interacting variables: outcome expectancy and efficacy. In this study, the results will signify the potential consequences of seeking or avoiding employment and admission information, while the effects reflect undergraduates' perceived ability to formulate and respond to their chosen information management decisions. The final phase of the theory, the decision, considers that information management behavior results from both direct and intermediary paths. These pathways include those between outcome expectancy and information management behaviors mediated by efficacy assessment and those between emotional responses and information management behaviors mediated by outcome expectancy and efficacy (Afifi and Morse, 2015). In our study, pathways A1, B1, B2, C, and D from the original TMIM theoretical framework were selected (Fowler and Afifi, 2011), and we hope to adhere to the theoretical basis model while further expanding it based on our findings.

Teachers positively impact students' learning engagement, their willingness to continue learning, and academic performance (Fang, 2023). They also provide essential support for students in campus activities, offering emotional, social, knowledge, and instrumental support (Li et al., 2019; Jiang et al., 2018; Liu et al., 2017; Malecki and Demaray, 2003). However, the guidance and service capabilities of teachers responsible for employment and further education in universities remain insufficient. The issues include a lack of employment guidance, a lack of growth interaction mechanisms, limited accumulation of professional capital after employment, and a lack of opportunities for offline training and learning (Li, 2024). Consequently, graduates often avoid engaging with their teachers when faced with these challenges. In addition, family members can provide informational support to graduates, making them feel more secure. This includes interactive communication, offering guidance, advice, information, and feedback (Liu, 2021). This support reflects mutual understanding, provides essential aid in students' studies and lives, and serves as psychological support within the family. A study suggests that individuals with high levels of psychological support are more capable of facing challenges and difficulties compared to those with low levels of psychological support (Barrera Jr, 1986). Additionally, friends are the most important source of social support for undergraduates. They are the main social relationships for individuals to obtain resources and assistance in the form of information, tools, and so on (Wang, 2020). The information support from friends is of considerable importance for graduating undergraduates. These three sources provide distinct types of informational support for undergraduates and cannot be discussed collectively. Therefore, this study focuses on teachers, family, and friends as communication subjects to investigate the discrepancies in information-seeking behavior among undergraduates when interacting with these groups.

### 2.1 Uncertainty discrepancy

Theory of Motivated Information Management (TMIM) explains that the decision to seek information is based on an individual's assessment and interpretation of the background in which the information exists. Therefore, the information management process begins with evaluating information uncertainty, defined as the uncertainty discrepancy, and individuals should consider emotions, possible outcomes, and effectiveness evaluations.

The discrepancy in uncertainty reflects how individuals perceive their level of uncertainty about a situation and how different the information they desire is from what they actually obtain in the information-seeking process. However, this uncertainty discrepancy is often ambiguous, complex, unpredictable, or probabilistic in various situations (Afifi and Weiner, 2004; Brashers, 2001). Meanwhile, uncertainty discrepancies may also arise when information is unavailable or inconsistent, or when individuals feel it lies beyond their knowledge domain. The uncertainty discrepancy is correlated with emotional responses, showing a positive correlation with negative emotions such as anxiety, fear, pain, and tension, and a negative correlation with positive emotions such as optimism, happiness, and pride (Rauscher and Hesse, 2014). After becoming aware of uncertainty levels that are higher or lower than expected and experiencing uncertainty-related anxiety, individuals assess both anticipated outcomes and their perceived ability to alleviate anxiety through information-seeking activities (Kuang and Wilson, 2021). Currently, Chinese undergraduates must not only keep up with the rapid changes in the digital transformation of social and economic forms and the accelerated development of social technology but also face the “devaluation of academic qualifications” caused by the expansion of higher education and the pressure of employment in a changing social and economic environment (Wu and Kong, 2024). Generally speaking, the discrepancies individuals notice during the interpretation phase lead to anxiety responses, and this uncertainty discrepancy results in varying levels of anxiety among individuals.

### 2.2 Anxiety

Anxiety can capture the varying degrees of psychological distress individuals experience during the process of information seeking, reflecting their emotional responses to uncertain employment information. Undergraduates' anxiety about employment and further education information can lead to a heightened state of pressure in which they are unable to make effective career decisions. Contributing factors include employment pressure, information quality, information literacy, and personal cognition in the processes of obtaining, screening, understanding, and using information (Mei and Liu, 2020). American scholar Wurman first proposed the concept of information anxiety in his book Information Anxiety in 1989 (Richard et al., 1989). He defined information anxiety as a state of stress that arises when individuals are unable to access, comprehend, or use the necessary information. It encompasses a range of complex psychological reactions triggered by both external and internal factors. External factors include information quality, retrieval effectiveness, and objective environment, while internal factors involve information literacy, personality traits, and attitudes toward information. These elements may include emotional responses, such as tension, anxiety, and fear, during the acquisition and application of information (Li and Cao, 2011). TMIM takes anxiety as the initial object of measurement. Afifi and Morse's research is based on the principles of emotional evaluation theory, using the Positive and Negative Emotion Schedule (PANAS). By expanding the TMIM to a wider range of emotional responses, it captures reactions such as anger, fear, disgust, jealousy, and hope (Rauscher and Hesse, 2014).

This study focuses on the correlation between uncertainty discrepancy and anxiety, both of which serve as interpretation stages for the TMIM. In the interpretation stage, when individuals find that their expected (non) level of certainty for an important issue is inconsistent with the actual situation, they believe that the discrepancy must be addressed. This discrepancy leads to anxiety related to uncertainty (Afifi and Weiner, 2004). Based on the above rationale, the TMIM framework should explain the mechanisms through which uncertainty motivates undergraduates' information-seeking about graduation. Specifically, there will be (including teachers, family, and friends as communication objects):

H1: Uncertainty discrepancy is positively associated with anxiety.
H2: Anxiety is negatively associated with outcome expectancy.
H3: Anxiety is negatively associated with efficacy.

### 2.3 Outcome expectancy

The outcome expectancy refers to the potential outcome of an individual's assessment of information management decisions, reflecting their consideration of the impact of implementing information management behavior. TMIM was used in a study on the search behavior of undergraduates regarding their partner's sexual history information (Afifi and Weiner, 2004). In the interpretation stage of TMIM, individuals evaluate potential outcomes (expected results) and their self-efficacy in seeking information. They determine whether to seek additional information based on two factors: whether the results are positive or negative, and whether the results are manageable. Positive outcome expectancy and high self-efficacy encourage individuals to engage in information-seeking behavior (Li et al., 2020).

### 2.4 Efficacy

Efficacy refers to an individual's confidence in their ability to perform specific behaviors and the belief that these behaviors or goals can lead to desired outcomes (Bandura, 1995, 1997). In TMIM, specific behaviors involve obtaining information from particular sources, while managing problematic outcomes helps reduce anxiety related to uncertainty. Similar to the initial evaluation phase, efficacy significantly influences an individual's final information management behavior. Efficacy consists of communication effectiveness (the ability to communicate effectively), coping effectiveness (the ability to handle potential outcomes), and target effectiveness (ensuring the integrity and honesty of the source) (Gettings and Kuang, 2021). Based on this rationale, various communication objects, including teachers, family, and friends, will be considered:

H4: Outcome expectancy is positive associated with efficacy.

### 2.5 Information seeking

Information seeking results from both direct and intermediary pathways. These pathways include connections between information-seeking, mediated by outcome expectancy and perceived efficacy, as well as relationships between emotional responses and information-seeking, also mediated by outcome expectancy and perceived efficacy (Afifi and Afifi, 2009).

H5: Efficacy is positively associated information seeking.

### 2.6 Proposed TMIM model

**Figure 2:**
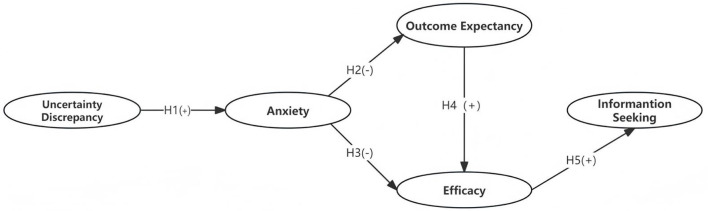
The Hypothesis TMIM model.

TMIM can explain the various information-seeking behaviors of undergraduates when confronted with information regarding their graduation destination and future choices. Therefore, this research proposes a hypothesis TMIM framework to understand the information decision-making processes and responses of undergraduates to different individuals, as shown in Figure 2. Based on the proposed framework, this study examines the TMIM models of information-seeking behaviors among undergraduates who have already graduated or are approaching graduation, focusing on interactions with teachers, family, and friends.

## 3 Methods

### 3.1 Participants

The participants included 310 individuals, with 35.9% men and 64.1% women. The majority of the respondents were between 18 and 25 years old (96.3%), followed by those aged 26 to 30 years (1.7%) and 31 to 40 (1.2%). The majority were undergraduates (89.4%), with a smaller portion having master's degrees or above (6.3%). The largest group of respondents were 4^*th*^-year students (56.1%), followed by 3^*rd*^-year (10.0%) and 2^*nd*^-year students (9.0%). More than half (55.5%) were taking postgraduate exams, and 52.4% participated in Spring Recruitment (spanning March to May, functioning as a supplementary phase targeting both delayed job-seekers) or Autumn Recruitment (occurring from September to November, serving as the primary hiring window for graduates in China). Regarding employment status, 20.6% had found a job, while 79.4% had not. The study sample was predominantly drawn from multiple universities across Eastern China, with participants from Guangdong Province constituting the largest proportion of the cohort. Concerning those students who were preparing for postgraduate exams, their participation in the examination did not necessarily indicate definitive career decisions, but rather reflected uncertainty in employment choices. In particular, some respondents had underperformed in the exam and subsequently transitioned to job seeking. When confronted with uncertain information, they demonstrated a tendency to systematically evaluate potential communication partners during their decision-making processes.

### 3.2 Procedures

The questionnaire was distributed through online platforms, primarily using social media channels such as Xiaohongshu, Weibo, WeChat, and QQ. A snowball sampling method was utilized to broaden the survey's reach, allowing for participant recruitment through existing networks. Initial participants were encouraged to share the questionnaire with their peers, which facilitated the collection of a more extensive and diverse dataset. To participate in the anonymous online survey, individuals had to meet three criteria: (a) be at least 18 years old, (b) be newly graduated or about to graduate, and (c) require relevant information. participated in an anonymous online survey. According to the theory of psychological distance, the experiences of students who graduated many years ago can influence individual decision-making concerning time, space, probability, and social distance (Jiang and He, 2017). Therefore, focusing on students who are facing graduation provided more accurate data. After consenting to participate, participants completed a questionnaire consisting of 86 questions, designed to measure uncertainty discrepancy, anxiety, outcome expectancy, and efficacy, as well as information-seeking behaviors with teachers, family, and friends. The minimum time required to complete the questionnaire was 180 s (3 min). The survey included two reverse-check questions to ensure data validity, and participants who did not answer these questions correctly were excluded from the final sample. Additionally, during the confirmatory factor analysis (CFA) phase of structural equation modeling (SEM) using AMOS, items with sub-threshold standardized factor loading (e.g., <0.5) and problematic multicollinearity were iteratively removed from the measurement models of individual latent variables to ensure construct validity and reliability.

### 3.3 Measures

All responses were 5-point Likert type scales (with higher values meaning more of the variable) unless otherwise noted.

#### 3.3.1 Uncertainty discrepancy

Uncertainty discrepancy regarding graduation information was measured using a modified version of Afifi et al. (2006). The revised questions adapted the original family-focused organ donation uncertainty scale to post-graduation planning contexts by emphasizing doubts about the reliability of information. Higher scores indicated a stronger degree of uncertainty. The scale was adapted to include five items, such as “I am not sure if my teachers, family members, or friends can provide me with relevant information,” and “I hope I can learn more about the thoughts and feelings about this information.”

#### 3.3.2 Anxiety

Anxiety was measured using a modified version of Afifi and Afifi (2009). The original questions examined anxiety stemming from knowledge gaps about parents' relationships, while the revised version reframes this anxiety within the context of post-graduation information gaps, ensuring alignment with the study's focus on graduates. Six items assessed the level of anxiety associated with uncertainty discrepancy, with statements such as “Do you feel anxious when you feel like you are not getting enough information? How anxious do you feel?”

#### 3.3.3 Outcome expectancy

Outcome expectancy was measured using the three-item index that previous TMIM tests have applied (Afifi et al., 2006), which is designed to understand participants' evaluation of the potential behavior of information management by the participants. Building on the original questions, the revised version adapted the context to address post-graduation information gaps, selectively modified items, and isolated negative expectations as a separate question. The index asked participants to consider statements like “Asking teachers/family members/friends for post-graduation information will bring me more benefits.” The index yielded high reliability.

#### 3.3.4 Efficacy

Efficacy was measured using a modified version of the Afifi and Morgan instrument (Afifi et al., 2006), which included six items such as “my teacher/family members/friends can provide me with post-graduation information..” and “I know how to communicate with my teachers/family members/friends to get the information I need.” The revised version modified the original items' wording and incorporated an additional item assessing perceived information completeness: “They possess relatively comprehensive information.”

#### 3.3.5 Information seeking

Information seeking was measured using a modified version of Fowler and Afifi's information management instrument (Fowler et al., 2018), which addressed both direct and avoidant information management. The items for direct information management included statements like “I have already discussed post-graduation issues with my teachers/family members/friends.” The avoidant information management items were reverse coded by SPSS Statistics 26 and included statements like “I do not want my teachers/family members/friends to give me information about post-graduation issues.” to test.

## 4 Results and findings

### 4.1 Evaluating the measurement model

The measurement model was evaluated through two steps. First, it was necessary to examine reliability and convergent validity for the three groups of data, and then discriminant validity was assessed next.

#### 4.1.1 Reliability and convergent validity

The composite reliability (CR) and average variance extracted (AVE) should be ≥0.7 (> 0.6 is acceptable) and ≥0.5 (>0.3 is acceptable), respectively. Fornell and Larcker believed that the AVE is not an absolute standard, and the scale can still have good aggregate validity when the AVE is slightly less than 0.5 and the composite reliability (CR) exceeds 0.6 (Fornell and Larcker, 1981). When the AVE value is greater than 0.36, this is an acceptable state, and >0.5 is the ideal state (Kline, 2023).

Table 1 shows the results regarding the teacher, where the CR values were more than 0.7, indicating that these items have high internal consistency and that the observed variables can reliably measure the latent variables. The AVE is between 0.467 and 0.751, which indicates that the model has acceptable convergent validity. The square multiple correlation (SMC) coefficients are mostly greater than 0.36 and less than 0.9, indicating the reliability of the items. The *p* values are all less than 0.001, suggesting significant effects between the factors.

**Table 1:** Measurement TMIM model (teacher).

**Constructs**	**Items**	**Significance estimation**				**Reliability**		**Composite reliability**	**Average variance extracted**
		**UnStd**	**S.E**.	**z-value**	*p*	**Std**.	**SMC**	**CR**	**AVE**
Uncertainty Discrepancy (teacher)	UDTe1	1				0.668	0.446	0.779	0.473
		UDTe 3	1.151	0.111	10.381	^***^	0.802			0.643		
		UDTe 4	1.08	0.109	9.88	^***^	0.716			0.513		
		UDTe 5	0.817	0.104	7.864	^***^	0.538			0.289		
Anxiety (teacher)	AnTe 1	1				0.693	0.48	0.792	0.495
		AnTe 3	1.038	0.096	10.859	^***^	0.76			0.578		
		AnTe 4	1.164	0.105	11.137	^***^	0.801			0.642		
		AnTe 7	0.757	0.095	7.967	^***^	0.526			0.277		
Outcome Expectancy (teacher)	OETe1	1				0.728	0.53	0.749	0.499
		OETe2	0.972	0.094	10.381	^***^	0.734			0.539		
		OETe3	0.925	0.097	9.567	^***^	0.654			0.428		
Efficacy (teacher)	EATe 1	1				0.74	0.548	0.724	0.467
		EATe4	1.073	0.109	9.84	^***^	0.664			0.441		
		EATe3	0.897	0.094	9.58	^***^	0.643			0.413		
Information Seeking (teacher)	IMTe1	1				0.853	0.728	0.9	0.751
		IMTe2	1.004	0.056	17.871	^***^	0.845			0.714		
		IMTe3	1.042	0.054	19.158	^***^	0.9			0.81		

Std., Factor loading; SMC, Std^2^, CR> 0.7 (> 0.6 acceptable), AVE> 0.5 (> 0.3 acceptable), ^***^p < 0.001.

Table 2 shows that, except for the uncertainty discrepancy (family), all variables have a CR of more than 0.7, demonstrating high internal consistency. The AVE ranges from 0.375 to 0.684, indicating acceptable convergent validity. The SMC coefficients, mostly greater than 0.36 and less than 0.9, indicate the reliability of the items. The *p* values are all less than 0.001, suggesting significant effects between the factors.

**Table 2:** Measurement TMIM model (family).

**Constructs**	**Items**	**Significance estimation**				**Reliability**		**Composite reliability**	**Average variance extracted**
		**UnStd**	**S.E**.	**z-value**	*p*	**Std**.	**SMC**	**CR**	**AVE**
Uncertainty Discrepancy (family)	UDFa5	1				0.601	0.361	0.643	0.375
		UDFa4	0.896	0.146	6.138	^***^	0.589			0.347		
		UDFa3	0.898	0.146	6.151	^***^	0.646			0.417		
Anxiety (family)	AnFa1	1				0.698	0.487	0.792	0.495
		AnFa3	1.031	0.095	10.803	^***^	0.759			0.576		
		AnFa4	1.155	0.105	11.033	^***^	0.801			0.642		
		AnFa7	0.75	0.095	7.934	^***^	0.525			0.276		
Outcome Expectancy (family)	OEFa1	1				0.833	0.694	0.808	0.591
		OEFa2	0.991	0.067	14.719	^***^	0.855			0.731		
		OEFa3	0.783	0.077	10.178	^***^	0.589			0.347		
Efficacy (family)	EAFa6	1				0.77	0.593	0.815	0.595
		EAFa5	0.973	0.081	12.065	^***^	0.762			0.581		
		EAFa4	0.921	0.075	12.265	^***^	0.782			0.612		
Information Seeking (family)	IMFa1	1				0.818	0.669	0.866	0.684
		IMFa2	1.082	0.068	15.908	^***^	0.887			0.787		
		IMFa3	0.986	0.069	14.331	^***^	0.773			0.598		

Std., Factor loading; SMC, Std^2^, CR> 0.7 (> 0.6 acceptable), AVE> 0.5 (> 0.3 acceptable), ^***^p < 0.001.

Table 3 shows the results regarding the friend, where the CR is greater than 0.6 for all variables, with most exceeding 0.7, demonstrating good internal consistency. The AVE ranges from 0.383 to 0.609, reflecting satisfactory convergent validity. Thus, the overall validity of the measurement model is confirmed by the standardized factor loadings. The SMC coefficients, mostly greater than 0.36 and less than 0.9, indicate the reliability of the items. The *p* values are all less than 0.001, suggesting significant effects between the factors.

**Table 3:** Measurement TMIM model (friend).

**Constructs**	**Items**	**Significance estimation**				**Reliability**		**Composite reliability**	**Average variance extracted**
		**UnStd**	**S.E**.	**z-value**	*p*	**Std**.	**SMC**	**CR**	**AVE**
Uncertainty Discrepancy (friend)	UDFr5	1				0.665	0.442		0.421
		UDFr4	0.965	0.149	6.479	^***^	0.712	0.507		0.674	
		UDFr1	0.713	0.11	6.462	^***^	0.535	0.286			
	Anxiety (friend)	AnFr1	1				0.695	0.483		0.792	0.495
		AnFr3	1.05	0.097	10.855	^***^	0.77	0.593				
		AnFr4	1.16	0.105	11.005	^***^	0.801	0.642				
		AnFr7	0.739	0.095	7.788	^***^	0.515	0.265				
Outcome Expectancy (friend)	OEFr1	1				0.528	0.279	0.647	0.383
		OEFr2	1.373	0.209	6.575	^***^	0.726			0.527		
		OEFr3	1.16	0.182	6.355	^***^	0.587			0.345		
	Efficacy (friend)	EAFr6	1				0.729			0.531	0.801	0.573
		EAFr5	1.15	0.099	11.568	^***^	0.789	0.623				
		EAFr3	1.085	0.096	11.244	^***^	0.751	0.564				
Information Seeking (friend)		IMFr1	1				0.798	0.637	0.834	0.609		
		IMFr2	1.036	0.081	12.856	^***^	0.815	0.664				
		IMFr3	0.933	0.078	11.972	^***^	0.726	0.527				

Std., Factor loading; SMC, Std^2^, CR> 0.7 (> 0.6 acceptable), AVE> 0.5 (> 0.3 acceptable), ^***^p < 0.001.

#### 4.1.2 Discriminant validity

Discriminant validity was tested for discriminatory validity among the reference variables. It was assessed by comparing the magnitude of the mean variance extracted with the correlation coefficient between the variables. If the correlation coefficient between a variable and other variables is less than the square root of the average variance extracted for that variable, the discriminant validity is considered good (Fornell and Larcker, 1981).

As shown in Tables 4–6, the bold font in the tables represents the square root of the extracted average variance, which is greater than all the values in the columns of the three tables. The figures indicate that the discriminant validity of the TMIM measurement model is appropriate for teachers, family members, and friends as information communication objects.

**Table 4:** The discriminant validity table of TMIM (teacher).

**Constructs**	**Convergence Validity**	**Discrimination validity**				
	**AVE**	**Information seeking (teacher)**	**Efficacy (teacher)**	**Outcome Expectancy (teacher)**	**Anxiety**	**Uncertainty discrepancy (teacher)**
Information seeking (teacher)	0.751	**0.867**				
Efficacy (teacher)	0.724	0.412	**0.851**			
Outcome expectancy (teacher)	0.499	0.593	0.814	**0.706**		
Anxiety (teacher)	0.495	0.088	0.118	0.096	**0.704**	
Uncertainty discrepancy(teacher)	0.473	–0.332	–0.25	–0.393	–0.505	**0.688**

The diagonal bold font is the AVE open root number value, and the lower triangle is the structural surface Pearson correlation coefficient.

**Table 5:** The discriminant validity table of TMIM (family).

**Constructs**	**Convergence validity**	**Discrimination validity**				
	**AVE**	**Information seeking (teacher)**	**Efficacy (teacher)**	**Outcome expectancy (teacher)**	**Anxiety**	**Uncertainty discrepancy (teacher)**
Information seeking (family)	0.684	**0.827**				
Efficacy (family)	0.595	0.487	**0.769**			
Outcome expectancy (family)	0.591	0.592	0.621	**0.711**		
Anxiety	0.495	0.002	0.059	0.068	**0.704**	
Uncertainty discrepancy(family)	0.375	-0.324	-0.431	-0.399	-0.226	**0.623**

The diagonal bold font is the AVE open root number value, and the lower triangle is the structural surface Pearson correlation coefficient.

**Table 6:** The discriminant validity table of TMIM (friend).

**Constructs**	**Convergence validity**	**Discrimination validity**				
	**AVE**	**Information seeking (teacher)**	**Efficacy (teacher)**	**Outcome expectancy (teacher)**	**Anxiety**	**Uncertainty discrepancy (teacher)**
Information seeking (friend)	0.609	**0.780**				
Efficacy (friend)	0.573	0.530	**0.757**			
Outcome expectancy (friend)	0.383	0.506	0.557	**0.619**		
Anxiety (friend)	0.495	–0.044	–0.200	–0.107	**0.704**	
Uncertainty Discrepancy(friend)	0.421	–0.234	–0.286	–0.169	–0.246	**0.649**

The diagonal bold font is the AVE open root number value, and the lower triangle is the structural surface Pearson correlation coefficient.

### 4.2 Structural models

Based on the above correlation analysis, this study established a TMIM structural equation model involving teachers, family members, and friends in information communication to understand how the same model adapts to different communication contexts by assessing the model fitting index. Specifically, it discusses whether the varying communication choices with teachers, family members, and friends influence the correlation of various variables in TMIM when encountering information related to graduation direction and aims to uncover the nature of their correlation.

Data analysis was conducted using AMOS26 for structural equation modeling (SEM) and pathway analysis, including multiple group comparisons. The fitting performance of the SEM was assessed by comparing the sample covariance matrix with that of the theoretical model, which generates a range of many model fit indices. Although AMOS provides 25 kinds of fit indices, only the most commonly used and relevant indicators were selected for reporting in this study.

The most common indicators include the chi-square value minimum difference (CMIN), the degrees of freedom (DF), the standardized chi-square value (CMIN/DF), the goodness of fit index (GFI), the adjusted goodness of fit index (AGFI), the comparative fit index (CFI), the non-normed fit index (TLI), the root mean square error of approximation (RMSEA), and the standardized root mean square residual (SRMR). In this study, the source of the assessment of the fit index refers to the opinions provided by authoritative experts in the field of structural equations, which refers to the Hayduck measurement standard of the standard card square value (CMIN/DF) (Hayduk, 1987); Bagozzi and Yi measurement criteria for the fit index (GFI), comparative fit index (CFI), adjusted fit index (AGFI), and root mean square error (RMSEA) (Bagozzi and Yi, 1988). The model fitting indicators are shown in Tables 7–9. Generally, GFI and CFI values greater than 0.5 are acceptable, and values greater than 0.9 are considered appropriate. RMSEA values less than 0.1 are acceptable, and those <0.08 are regarded as good. Therefore, the most fitting model was the TMIM model with friends (as shown in Table 9). The results indicated a good fit to the model with χ^2^/*df* = 2.189, CFI = 0.919, GFI = 0.919, and RMSEA = 0.063. In addition, the results of the structural model for the TMIM model with teachers are illustrated in Table 7, which suggested a good model fit (χ^2^/*df* = 2.428, CFI = 0.920, GFI = 0.906, RMSEA = 0.069). Similarly, the results of the structural model for the TMIM model with family (Table 8) suggested a good fit to the model, where χ^2^/*df* = 3.218, CFI = 0.886, GFI = 0.882, and RMSEA = 0.086. The model fitting data in this paper are ideal; thus, subsequent hypothesis verification can be conducted.

**Table 7:** Model fitting indicators (teachers).

**Metric**	**Model index value**	**Standard**	**Conclusion**
CMIN/DF	2.428	<3 Excellent; <5 is Acceptable	Excellent fit
GFI	0.906	>0.8 Acceptable; >0.9 Good fit	Good fit
AGFI	0.874	>0.8 Acceptable; >0.9 Good fit	Acceptable
CFI	0.920	>0.8 Acceptable; >0.9 Good fit	Good fit
TLI(NNFI)	0.904	>0.8 Acceptable; >0.9 Good fit	Good fit
RMSEA	0.069	<0.08 Excellent; <0.1 is Acceptable	Excellent fit

**Table 8:** Model fitting indicators (family).

**Metric**	**Model index value**	**Standard**	**Conclusion**
CMIN /DF	3.218	<3 Excellent; <5 is Acceptable	Acceptable
GFI	0.882	>0.8 Acceptable; >0.9 Good fit	Acceptable
AGFI	0.838	>0.8 Acceptable; >0.9 Good fit	Acceptable
CFI	0.886	>0.8 Acceptable; >0.9 Good fit	Acceptable
TLI(NNFI)	0.862	>0.8 Acceptable; >0.9 Good fit	Acceptable
RMSEA	0.086	<0.08 Excellent; <0.1 is Acceptable	Acceptable

**Table 9:** Model fitting indicators (friend).

**Metric**	**Model index value**	**Standard**	**Conclusion**
CMIN /DF	2.189	<3 Excellent; <5 is Acceptable	Excellent fit
GFI	0.919	>0.8 Acceptable; >0.9 Good fit	Good fit
AGFI	0.889	>0.8 Acceptable; >0.9 Good fit	Acceptable
CFI	0.919	>0.8 Acceptable; >0.9 Good fit	Good fit
TLI(NNFI)	0.902	>0.8 Acceptable; >0.9 Good fit	Good fit
RMSEA	0.063	<0.08 Excellent; <0.1 is Acceptable	Excellent fit

### 4.3 Structural evaluation

**Figure 3:**
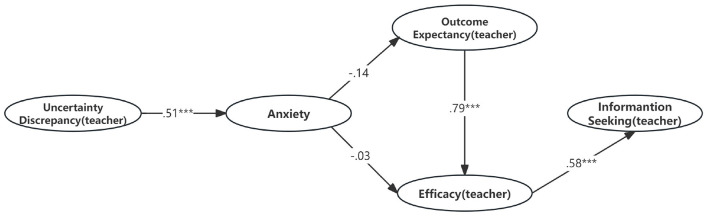
The TMIM with standardized path coefficients (teacher). ***p < 0.001.

The results in Table 10 and Figure 3 indicated that the uncertainty discrepancy (teacher) positively predicted anxiety (β = 0.514, *p* < 0.05), but anxiety did not negatively predict the outcome expectancy (teacher) and efficacy (teacher). The outcome expectancy (teacher) positively predicted efficacy (teacher) (β = 0.79, *p* < 0.05). Consistent with our expectations, efficacy (teacher) positively predicted information seeking (teacher) when referring to the teacher (β = 0.575, *p* < 0.05). In addition, outcome expectancy (teacher) and anxiety jointly explained 63.3% of the variance.

**Table 10:** Results of hypothesis testing of teacher.

**Relationship**				**UnStd**.	**S.E**.	**C.R**.	***p***	**Std. (β)**	**R2**	**Supported**
H1	Uncertainty discrepancy (teacher)	→	Anxiety	0.459	0.072	6.337	***	0.514	0.264	Yes
H2	Anxiety	→	Outcome expectancy (teacher)	-0.142	0.075	-1.887	0.059	-0.139	0.019	No
H3	Anxiety	→	Efficacy (teacher)	-0.033	0.057	-0.573	0.567	-0.035	0.633	No
H4	Outcome expectancy (teacher)	→	Efficacy (teacher)	0.724	0.082	8.831	^***^	0.79			Yes
H5	Efficacy (teacher)	→	Information Seeking (teacher)	0.967	0.121	7.973	^***^	0.575	0.331	Yes

***p < 0.001.

**Figure 4:**
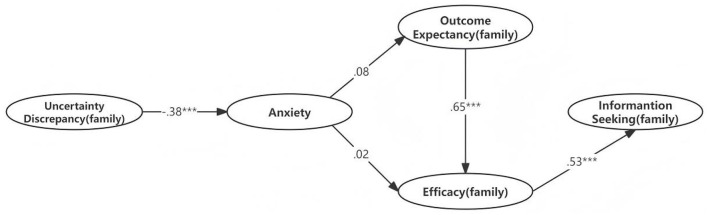
The TMIM with standardized path coefficients (family). ^***^p < 0.001.

The results in Table 11 and Figure 4 indicated that the uncertainty discrepancy (family) negatively predicted anxiety (β = −0.378, *p* < 0.05) but anxiety did not negatively predict the outcome expectancy (family). The outcome expectancy (family) had a positive effect on efficacy (β = 0.654, *p* < 0.05). Consistent with our expectations, efficacy (family) positively predicted information seeking (family) (β = 0.53, *p* < 0.05). Outcome expectancy (family) and anxiety together explained 43.1% of the variance.

**Table 11:** Results of hypothesis testing of family.

**Hypothesis**	**Relationship**			**UnStd**.	**S.E**.	**C.R**.	***p***	**Std. (β)**	**R2**	**Supported**
H1	Uncertainty discrepancy (family)	→	Anxiety	–0.346	0.083	–4.193	^***^	–0.378	0.143	No
H2	Anxiety	→	Outcome expectancy (family)	0.103	0.086	1.191	0.234	0.084	0.007	No
H3	Anxiety	→	Efficacy (family)	0.028	0.072	0.383	0.702	0.022	0.431	No
H4	Outcome expectancy (family)	→	Efficacy (family)	0.657	0.072	9.067	^***^	0.654			Yes
H5	Efficacy (family)	→	Information Seeking (family)	0.592	0.075	7.942	^***^	0.53	0.281	Yes

^***^p < 0.001.

**Figure 5:**
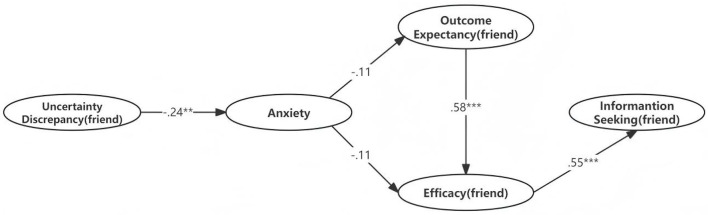
The TMIM with standardized path coefficients (friend). ^**^p < 0.01, ^***^p < 0.001.

Table 12 and Figure 5 indicate that the uncertainty discrepancy (friend) does not positively predict anxiety. Furthermore, anxiety is not associated with outcome expectancy (friend) and efficacy (friend), indicating that the model cannot confirm the theoretical interpretation of the TMIM at the evaluation stage. However, the expected outcome (friend) is positively associated with efficacy (friend) (β = 0.578, *p* < 0.05), and efficacy (friend) positively predicts information seeking (friend) (β = 0.553, *p* < 0.05). Together, the expected outcome (friend) and anxiety explain 36.2% of the variance.

**Table 12:** Results of hypothesis testing of friend.

**Hypothesis**	**Relationship**			**UnStd**.	**S.E**.	**C.R**.	***p***	**Std. (β)**	**R2**	**Supported**
H1	Uncertainty Discrepancy (friend)	→	Anxiety	–0.209	0.069	–3.033	0.002	–0.244	0.06	No
H2	Anxiety	→	Outcome Expectancy (friend)	–0.072	0.053	–1.371	0.17	–0.108	0.012	No
H3	Anxiety	→	Efficacy (friend)	–0.103	0.059	–1.731	0.084	–0.114	0.362	No
H4	Outcome Expectancy (friend)	→	Efficacy (friend)	0.779	0.138	5.663	^***^	0.578			Yes
H5	Efficacy (friend)	→	Information Seeking (friend)	0.697	0.094	7.39	^***^	0.553	0.305	Yes

^***^p < 0.001.

### 4.4 Discussion and conclusion

The results indicated that anxiety among undergraduates due to graduation was generally not influenced by factors related to information uncertainty. The TMIM model of seeking friends as objects could not support H1, which may be attributed to undergraduates possessing higher levels of interpersonal efficacy and peer support, which helped them enhance their self-confidence and foster more positive emotions (Chen, 2020). When faced with difficulties or uncertain situations in life, they are able to analyze rationally and solve problems. Additionally, there was a negative association between family-related uncertainty and anxiety, which also failed to support H1.

In the evaluation stage, models that sought information from teachers, family members, and friends could not generally support H2 and H3, and the negative emotions generated by undergraduates due to information uncertainty could not predict the lower outcome expectancy and efficacy. This result indicated that undergraduates were not affected by excessive anxiety when faced with uncertain information. Teachers, family members, and friends could provide the necessary informational support for undergraduates when faced with graduation decisions. This was related to the dual role that families played in the growth process of undergraduates. Family members could provide multiple forms of support, including informational support, for undergraduates, contributing important factors to the growth environment. Thus, with family members, undergraduates could create a harmonious family atmosphere and healthy family values during communication. The results also indicated that undergraduates could fully trust their teachers to provide the necessary information. Moreover, existing research has also proven that friends are the most important source of social support for undergraduates (Chen, 2020). When dealing with stress, people also receive several different types of support, including informational support, which involves the provision of facts, advice, or perspective (High and Crowley, 2018).

In addition, for negative outcomes, they tended to solve them on their own rather than seeking help. This may relate to the current information literacy of undergraduates. Once they learn that they can independently obtain relevant information, they will worry less about handling negative responses from teachers, family members, and friends. In the TMIM decision stage, models that sought information from teachers, family members, and friends could support H4 and H5. The results of H4 showed an expected positive prediction of their perception of communication effectiveness. H5 indicated that the positive association between efficacy and information seeking was predicted, revealing that the more skilled the coping and communication abilities of the undergraduates were, the more likely they were to engage in seeking graduation-related information.

Above all, it could reflect that when faced with information discrepancies about the future upon graduating, undergraduates were able to seek help with enthusiasm. At the same time, they paid more attention to the teacher's views on graduation-related information and hoped to obtain more comprehensive information. Furthermore, when faced with the uncertainty of information, friends were more familiar with and comfortable approaching them. When they obtained effective opinions from their friends, it could encourage them to take different information management actions.

The uncertain management of undergraduates' graduation direction information is usually affected by the object of information seeking. This study proposed hypotheses based on the TMIM but did not support all of them. This suggests that it still has significant limitations. The results confirm the effectiveness of the TMIM framework in predicting information-seeking strategies and support the application of undergraduates' information-seeking patterns in various scenarios.

However, research needs further improvement. (1) Since data are sent and filled out online, the validity of the data cannot be guaranteed, and subsequent research needs to carry out more accurate questionnaire distribution. (2) In explaining the hypothesis, the TMIM model shows no significant correlation among anxiety, outcome expectancy, and efficacy, and the investigation needs to enhance data collection and analysis. (3) The questionnaire survey must be expanded to collect sufficient data. (4) There are no similar results from the TMIM study for comparison with the results of this study, and no further research support can be provided. In summary, future research will also address the above issues more effectively.

## Data Availability

The raw data supporting the conclusions of this article will be made available by the authors, without undue reservation.
